# Hydrogen peroxide damage to rat liver sinusoidal endothelial cells is prevented by *n*-acetyl-cysteine but not GSH

**DOI:** 10.1097/HC9.0000000000000617

**Published:** 2025-01-16

**Authors:** Larissa D. Kruse, Christopher Holte, Bartlomiej Zapotoczny, Eike C. Struck, Jasmin Schürstedt, Wolfgang Hübner, Thomas Huser, Karolina Szafranska

**Affiliations:** 1 Vascular Biology Research Group, Department of Medical Biology, University of Tromsø—The Arctic University of Norway, Tromsø, Norway; 2 Institute of Nuclear Physics Polish Academy of Sciences, Krakow, Poland; 3 Translational Vascular Research Group, Department of Clinical Medicine, University of Tromsø—The Arctic University of Norway, Tromsø, Norway; 4 Biomolecular Photonics Research Group, Department of Physics, Bielefeld University, Bielefeld, Germany

**Keywords:** GSH, LSEC, NAC, oxidative stress, ROS

## Abstract

**Background:**

Reactive oxygen species (ROS) are prevalent in the liver during intoxication, infection, inflammation, and aging. Changes in liver sinusoidal endothelial cells (LSEC) are associated with various liver diseases.

**Methods:**

Isolated rat LSEC were studied under oxidative stress induced by H_2_O_2_ at different concentrations (0.5–1000 µM) and exposure times (10–120 min). LSEC functions were tested in a dose-dependent and time-dependent manner.

**Results:**

(1) Cell viability, reducing potential, and scavenging function decreased as H_2_O_2_ concentration and exposure time increased; (2) intracellular ROS levels rose with higher H_2_O_2_ concentrations; (3) fenestrations exhibited a dynamic response, initially closing but partially reopening at H_2_O_2_ concentrations above 100 µM after about 1 hour; (4) scavenging function was affected after just 10 minutes of exposure, with the impact being irreversible and primarily affecting degradation rather than receptor-mediated uptake; (5) the tubulin network was disrupted in high H_2_O_2_ concentration while the actin cytoskeleton appears to remain largely intact. Finally, we found that reducing agents and thiol donors such as *n*-acetyl cysteine and glutathione (GSH) could protect cells from ROS-induced damage but could not reverse existing damage as pretreatment with *n*-acetyl cysteine, but not GSH, reduced the negative effects of ROS exposure.

**Conclusions:**

The results suggest that LSEC does not store an excess amount of GSH but rather can readily produce it in the occurrence of oxidative stress conditions. Moreover, the observed thresholds in dose-dependent and time-dependent changes, as well as the treatments with *n*-acetyl cysteine/GSH, confirm the existence of a ROS-depleting system in LSEC.

## INTRODUCTION

Oxidative stress can be defined as an imbalance between production, accumulation, and elimination of reactive oxygen species (ROS). ROS is a collective term for certain oxygen-containing oxidizing compounds, including but not limited to oxygen radicals. Radical ROS includes superoxide (O_2_^−•^), hydroxyl (HO^•^), and peroxyl radicals (HOO^•^), while nonradical ROS includes peroxide, hypochlorous acid, and ozone.^1^ In biology, the role of ROS creates a paradox where the thin line between toxic and physiological effects is constantly being shifted. In homeostasis, ROS are present both intracellularly and extracellularly and can act as signaling molecules.^2^ The respiratory chain, lipoxygenases/cyclooxygenases, NO-synthesis, nicotinamide adenine dinucleotide oxidases, and xanthine oxidase are the main sources of intracellular ROS.^3^ On the other hand, oxidative stress is associated with the pathogenesis of many diseases, aging, promotion of inflammation, and cytotoxicity.^1^,^4^ The redox state balance in the cells is kept by defense systems depending on enzymatic components, such as superoxide dismutase, catalase, and glutathione (GSH) peroxidase, which protect cells from ROS-induced cellular damage.^5^

In the liver, ROS can be produced through endogenous (eg, mitochondria, ER, and peroxisomes) and exogenous (eg, heavy metals and pollutants) sources.^6^ Oxidative stress is implicated in pathogeneses of diseases, DILI, reperfusion injury, sinusoidal obstruction syndrome, but also in inflammatory responses and aging.^4^,^7^ Even in nonpathological states, due to its location in the cardiovascular system, the liver is constantly under oxidative stress, with portal blood and highly metabolically active hepatocytes being the main ROS sources.^8^ The portal vein is the main blood supply of the liver and the source of gut-derived toxins and other substances absorbed via the gastrointestinal tract. Our research underscores vital yet previously understudied aspects of liver physiology—specifically, the unique susceptibility and response of LSEC to oxidative stress. While the role of ROS in liver pathology is well-documented, our findings shed new light on the critical position of LSEC within this framework. Positioned strategically between the 2 primary sources of ROS in the liver—blood flow and hepatocytes—LSEC not only serves as a barrier but also as a regulator of crucial transport processes through the space of Disse.^9^,^10^ This unique anatomical and functional placement subjects them to an intense oxidative milieu, distinct from other liver cell types. Our study, therefore, not only expands the understanding of LSEC roles under oxidative conditions but also delineates their critical involvement in the liver’s overall response to oxidative stress, which could pave the way for targeted therapeutic interventions. This is particularly novel, as these cells, while constituting only about 15%–20% of the total number of liver cells and comprising just 3% of the liver volume, often get overshadowed by hepatocytes, which constitute ~60% of the number of liver cells and make up about 80% of liver volume.

Since LSECs are especially vulnerable to ROS stimuli, a defensive GSH-based system was proposed that allows maintenance of the redox balance under physiological conditions.^11^,^12^ To deepen the knowledge about the LSEC and ROS interaction, we investigated the time-dependent and dose-dependent effects of H_2_O_2_ on rat LSEC morphology and functions in vitro. Moreover, by using the pretreatment and cotreatments with ROS-depleting agents *n*-acetyl cysteine (NAC) and GSH, we further study anti-ROS defense mechanisms in LSEC.

## METHODS

### LSEC isolation and cell culture

LSEC were isolated from male Sprague-Dawley rats using the modified protocol described in Smedsrød and Pertoft.^13^ Samples were incubated for 3 hours in RPMI-1640 media at 37 °C with 5% CO_2_/5% O_2_ before treatments (details in Supplemental Section S1, http://links.lww.com/HC9/B868).

### Viability assays

#### Lactase dehydrogenase

LSECs were seeded on 48-well plates (300,000 cells/well), and a luminescence lactase dehydrogenase (LDH) detection kit (LDH-Glo, Promega) was used following the manufacturer’s instructions to assess cell viability. After treatments, 50 µL of media samples were collected at selected time points (0.5–5 h) into 450 µL of freezing buffer (details in the manufacturer’s protocol) and stored at −20 °C until measurements.

#### Resazurin

LSECs were seeded on 48-well plates (300,000 cells/well), and a Resazurin/resorufin assay (Biotechne) was used as an indicator of mitochondrial function and viability. Together with the treatments, 1:10 resazurin reagent was added to the culture media. After time points were set (1, 2, 3, and 4/5 h), supernatants (50 µL) were collected, and fluorescence was measured (excitation 530–570 nm and emission 580–590 nm).

### Scavenging assay

^125^I radiolabeled formaldehyde-treated serum albumin (FSA) was used for quantitative studies of endocytosis in LSEC. Thirty nanograms of ^125^I-FSA were added to each well and incubated for 2 hours. Thereafter, the cell-associated and degraded FSA fractions were analyzed (details in Supplemental Section S2, http://links.lww.com/HC9/B868).

### Imaging

The detailed methods of sample preparation and imaging for light and electron microscopy are described in Szafranska et al.^14^ and Supplemental Section S3, http://links.lww.com/HC9/B868. Image analysis protocols and statistics can be found in Supplemental Section S4, http://links.lww.com/HC9/B868. Rat LSECs were seeded on fibronectin-coated well plates with a density of about 60,000 cells/well and, after treatments, fixed using McDowell’s solution (4% formaldehyde and 1% glutaraldehyde) for EM and 4% formaldehyde for light microscopy. EM samples were treated with 1% tannic acid, 1% osmium-tetroxide, dehydrated in an ethanol gradient (30%→100%), chemically dried in hexamethyldisilazane, and coated with a 10 nm layer of gold/palladium alloys.

Structured illumination microscopy/fluorescence microscopy samples were permeabilized with 0.5% Triton-X100 for 90–120 seconds and stained with anti-α-tubulin antibodies-AlexaFluor647, Phalloidin-AlexaFluor555, and DAPI. Samples were mounted with ProLong-glass until imaging with widefiled fluorescence microscopy or structured illumination microscopy. Quantitative analysis of LSEC tubulin cytoskeleton was performed using open access software FilamentSensor2.0^15^ according to the protocol introduced previously in Czyzynska-Cichon et al.^16^ Examples of the analyzed images are presented in Supplemental Figure S4, http://links.lww.com/HC9/B868.

For atomic force microscopy, LSECs were isolated and cryopreserved as described previously in Mönkemöller et al.^17^ All measurements of cell dynamics were performed using Nanowizard4 (JPK Instruments) in Quantitative Imaging mode in 37 °C/EGM-2 media according to the methodology described before.^14^,^18^ The loading force used for QI measurements ranged from 0.2 to 0.3 nN and was adjusted to the scanning conditions for individual silicon nitride cantilevers (SCM-PIC-V2, Bruker) characterized by a nominal spring constant of 0.1 N/m and a nominal tip radius of 25 nm. Three independent experiments were conducted.

### ROS detection assay

A commercially available ROS indicator was used for the detection of intracellular ROS (CM-H2DCFDA, Invitrogen) according to the manufacturer’s protocol. LSEC media was exchanged to HBSS containing 3 µg/mL of the dye and incubated for 45 minutes at 37 °C (without CO_2_). Afterward, cells were rinsed and incubated for an additional 20 minutes in RPMI before treatment with selected agents. Fluorescent images were taken from live cells in HBSS directly after 60-minute treatments and analyzed using ImageJ/Fiji^19^ to compare fluorescence intensity signals.

## RESULTS

### Effects of ROS on LSEC scavenging function and viability

**FIGURE 1:**
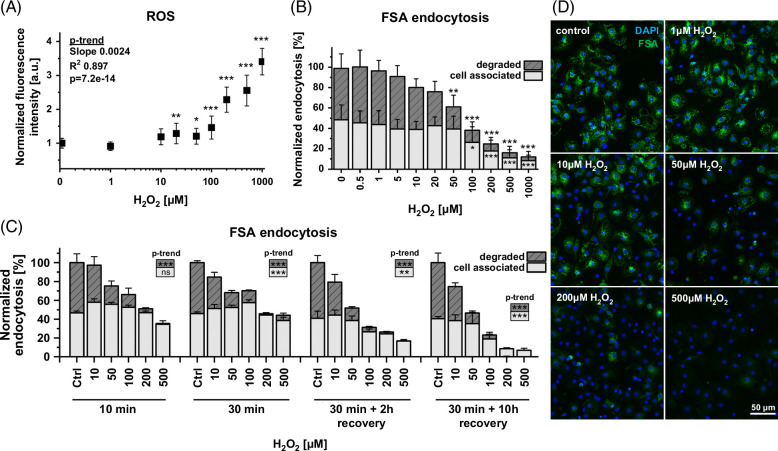
Effects of ROS on endocytosis of trace amount of FSA in rat LSEC. (A) Intracellular ROS level after 1 hour of treatment with 0–1000 µM hydrogen peroxide. Fluorescence-based assay data were normalized to the untreated control to show the fold-increase. Mean ± SD. (B, C) The effects of H_2_O_2_ on scavenging of ^125^I-FSA in LSEC. The total endocytic activity was 40%–60% of the added radioactivity and was normalized to the untreated control. (B) Cells were treated with hydrogen peroxide together with ~30 ng/mL of radiolabeled FSA for 2 hours. (C) Cells were treated for 10/30 minutes with hydrogen peroxide, then rinsed and either directly incubated with ~30 ng/mL of radiolabeled FSA for 2 hours or recovered with RPMI media for 2 hours or 10 hours before 2-hour incubation with radiolabeled FSA. Dark/striped bars represent a degraded fraction, while light bars represent a cell-associated fraction of the ligand (±SD), (A/C) n = 3–4 bioreplicates, (B) n = 7. (D) Uptake of FSA-AF488 after treatment with hydrogen peroxide shows a heterogeneous population of LSEC. FSA was added for the last 15 minutes of the 2-hour treatment with H_2_O_2_. Significance indicated in relation to the control (A, B) or as p-for-trend analysis (A, C), ns, not significant, *p < 0.05, **p < 0.01, ***p < 0.001, details in Supplemental Tables S1–3, http://links.lww.com/HC9/B868. Abbreviations: FSA, formaldehyde-treated serum albumin; ROS, reactive oxygen species.

First, a concentration range of the ROS-inducing factor hydrogen peroxide was tested to establish the amount necessary to change intracellular ROS levels in LSEC. A fluorescent-based ROS detection assay was used on LSEC challenged with increasing concentrations of H_2_O_2_ (Figure 1A). No detectable ROS increase was observed for H_2_O_2_ concentrations below 5 µM and a small elevation of ROS was detected for medium concentrations (5–50 µM H_2_O_2_). A dose-dependent increase was observed for high (100–1000 µM) concentrations of H_2_O_2_, reaching a 3.5-fold increase at 1000 µM.

The ROS effect on the scavenging system was studied using FSA assays (Figures 1B–D). FSA is a ligand for stabilin-1 and -2^20^ and allows for assessment of both uptake and degradation by the LSEC scavenging system.^21^ Low concentrations of hydrogen peroxide (<5 µM) did not affect scavenging, while in concentrations of 5–50 µM a steady decrease in the degraded fraction of FSA was observed, up to a 40% reduction (total scavenging activity was significantly reduced, *p*_tot_ = 1.43E−09 [***]). For high concentrations of hydrogen peroxide (>100 µM), a significant drop in degraded and cell-associated fractions of FSA occurred with almost complete inhibition of degradation at 1000 µM H_2_O_2_ (statistical analyses in Supplemental Table S2, http://links.lww.com/HC9/B868). Linear model analysis revealed a significant downward shift for degraded (*p*_degraded_ = 1.00E−09 [***]) and cell-associated (*p*_cell_associated_ = 2.57E−08 [***]) fractions over increased doses of H_2_O_2_. We applied a split model to determine the effect threshold. The linear model for degraded and cell-associated values was split into doses from 0–50 µM H_2_O_2_ and 50–1000 µM. The latter showed a significant reduction for both models (*p*_degraded_50–1000_ = 0.009 [**], *p*_cell_associated_50-1000_ = 0.0005 [***]), while 0–50 µM H_2_O_2_ was significant only in the degraded fraction (*p*_degraded_0–50_ = 2.59E−06 [***]), showing a stronger effect at higher concentrations of H_2_O_2_ with more influence on the degradation than uptake (results in Supplemental Table S1, http://links.lww.com/HC9/B868). The effect on scavenging in different concentration ranges of hydrogen peroxide resembles the detected intracellular ROS levels. Similar trends were observed with a scavenging assay based on radiolabeled collagen-α-chain, a specific ligand of mannose receptor (Supplemental Figure S1, http://links.lww.com/HC9/B868).

These results were confirmed in qualitative experiments using fluorescently labeled FSA, where endocytosis ligands were added for the last 15 minutes of the 2-hour treatment with H_2_O_2_ (Figure 1D). No FSA uptake was observed for concentrations above 500 µM, while for concentrations of 50–200 µM only a fraction of the LSEC population showed remaining endocytic activity. This finding suggests that the decrease in scavenging function is a result of a decreased number of cells that can efficiently perform endocytosis rather than lower endocytic activity per cell.

To understand the dynamics and reversibility of the effect, different durations of ROS induction in LSEC were studied using the quantitative/radiolabeled scavenging assay (Figure 1C). First, LSECs were treated with different doses of H_2_O_2_ for 10/30 minutes before the addition of FSA. Both treatments showed a decrease in the endocytic activity for H_2_O_2_ concentrations above 10 µM. In addition, we investigated whether scavenging systems remained irreversibly damaged by applying recovery times of either 2 hours or 10 hours after the initial challenge with H_2_O_2_ in multiple concentrations. The results suggest that scavenging systems remain irreversibly damaged despite the recovery time, and a further decrease in FSA uptake was observed 10 hours after the initial treatment. The linear model shows a significant reduction in degraded fractions over all experiments (Figure 1C). The positive slope of the significant cell-associated shift suggests it is driven by the upward trend in low concentrations of of H_2_O_2_ (Supplemental Table S3, http://links.lww.com/HC9/B868).

**FIGURE 2:**
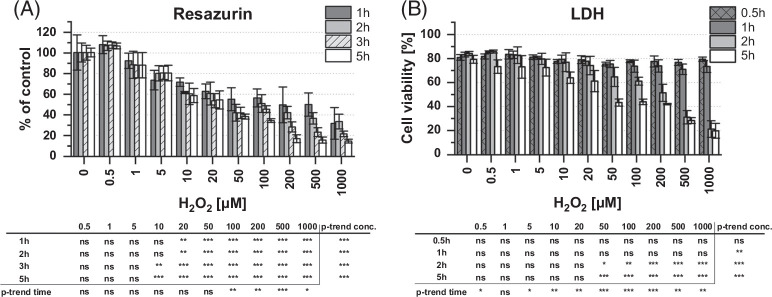
Influence of hydrogen peroxide–induced ROS on rat LSEC viability. Functional viability/reducing potential of the cell was assessed using resazurin/resorufin assay (A) and structural viability/cell integrity was studied with LDH-release assay (B). Measurements were conducted under continuous treatment with hydrogen peroxide on selected time points (0.5–5 h). The cell viability was calculated and normalized to the untreated control for resazurin assay and to the total LDH after cell lysis for LDH-release assay. Average data from 3 independent experiments/bioreplicates ±SD are presented. Statistical analyses below graphs show *p* values for trend analysis in time and concentration dependency. ANOVA for different times and concentrations compared to control values of nontreated cells; * p<0.05, ** p<0.01, *** p<0.001, ns non-significant. Abbreviations: LDH, lactase dehydrogenase; ROS, reactive oxygen species.

The changes in measured scavenging activity can be a result of either disruption of the scavenging system or damage to the cell resulting in cell death. Two approaches of viability assays were used for confirmation. Functional viability was assessed using the resazurin assay (Figure 2A), while structural integrity was studied using LDH release viability assay (Figure 2B).

Resazurin assay measurements correspond with the reducing power of the cell, often connected to mitochondrial metabolic activity. A significant dose-dependent decrease was observed for all time points for concentrations in the range of 20–1000 µM, while a time-dependent decrease occurred for concentrations of 100 µM H_2_O_2_ and higher. For treatments in the range of 0.5–100 µM H_2_O_2_, the initial decrease observed after 1 hour of treatment did not progress further. For concentrations of **≥**100 µM H_2_O_2_, the initial effect after 1 hour was followed by further progressive reduction until reaching below 20% of the control after 5 hours of treatment.

Upon cell membrane damage, LDH is released into the culture medium where it can be quantitatively measured. It describes the membrane integrity and can indicate structural viability. Initial structural viability changes in untreated cells are related to cell death occurring during the early hours after isolation of LSECs. Treatment with hydrogen peroxide did not affect structural viability in the first hour at any concentration. However, after 2 and 5 hours of treatment with H_2_O_2_, a significant dose-dependent increase in LDH release compared to control was observed for treatments with 50 µM and above.

Both results suggest that cells in low H_2_O_2_ concentrations are affected rapidly after exposure but remain stable afterward without further damage. Functional and structural viability progressively decreased in time in concentrations >50 µM of H_2_O_2_. This pattern indicates the existence of a ROS-depleting system in LSEC that can mitigate effects of ROS until a concentration threshold.

### ROS-induced changes in LSEC morphology

**FIGURE 3:**
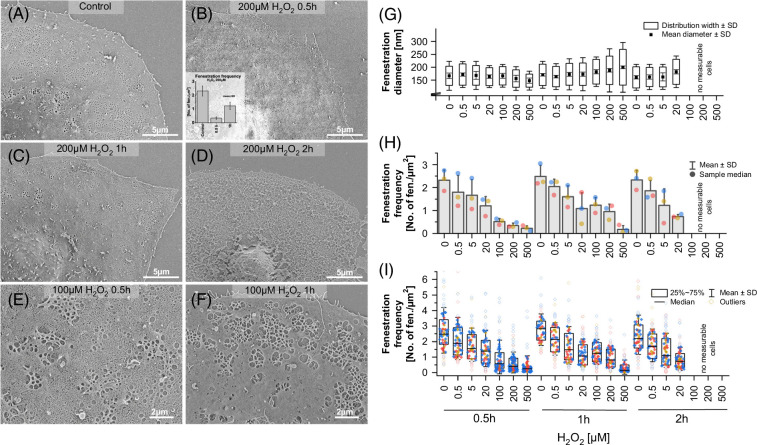
Changes in LSEC fenestrated morphology after exposure to H_2_O_2_. (A–F) Representative scanning electron microscopy images and (G–I) quantitative analysis results of hydrogen peroxide–treated rat LSEC. A clear dose-dependent effect was observed for all time points (0.5–2 h). Treatment with 100–200 μM H_2_O_2_ resulted in an initial decrease in fenestration number after 0.5 hours, followed by an increase in fenestration number after 1 hour in a part of the cell population and later degradation of the cell membrane after 2 hours of treatment. (G–I) SEM images of rat LSEC treated with 0–500 µM H_2_O_2_ for 0.5–2 hours were analyzed to obtain parameters such as fenestration diameter, fenestration frequency, and porosity (Supplemental Figure S2, http://links.lww.com/HC9/B868). (G) Mean ± SD was calculated from medians of fenestration diameters for each sample and distribution widths were calculated at the half maximum of the Gaussian distribution fit. (H) Each dot represents the mean fenestration frequency value calculated from each bioreplicate (all data points shown in (I)). (I) Each point represents data from a single cell, and each color is an individual bioreplicate. Abbreviation: SEM, scanning electron microscopy.

Effects of ROS on LSEC morphology were studied using microscopy. The detailed morphological structure was observed with scanning electron microscopy (SEM) (Figures 3A–F), and images were quantitatively analyzed to calculate fenestration diameter, frequency, and porosity (Figures 3G–I, Supplemental Figure S2, http://links.lww.com/HC9/B868). For all time points, a significant, dose-dependent reduction in the number of fenestrations was observed (*p* for trend: *p*_0.5 h_ = 0.0002 [***]; *p*_1 h_ = 4.59E−05 [***]; *p*_2 h_ = 0.0013 [**], Supplemental Table S5, http://links.lww.com/HC9/B868). In addition, distorted sieve plates resembling previously reported defenestration centers (DFC)^18^ were observed for H_2_O_2_ concentrations above 5 µM (Figures 3B, C). After the first 0.5 hours of treatment, near complete defenestration was observed for 100–500 µM H_2_O_2_ while after 1 hour of treatment, the number of fenestrations increased, however never returning to control levels (Figures 3B, H). Significant, biologically relevant differences in fenestration frequency could be observed between the control and 500 µM H_2_O_2_, for 1 hour and between 30 minutes and 1 hour of treatment of 500 µM H_2_O_2_ (*p* values in Supplemental Table S4, http://links.lww.com/HC9/B868). The fenestration frequency data show that the cell population became heterogeneous with some cells remaining defenestrated while others regained porous morphology (Figure 3I). For high (>100 µM) H_2_O_2_ concentrations, nearly no viable cells were observed after 2 hours, with a majority of the sample presenting distorted/discontinued cell membranes, suggesting necrotic cell death (Figure 3D). This observation confirms the previous structural viability data where a significant increase in the LDH release was detected only after 2 hours of treatment with >100 µM H_2_O_2_ with no significant increase for 0.5 and 1 hour (Figure 2B). In samples treated with H_2_O_2_ concentrations below 20 µM, a dose-dependent loss of fenestrations was observed for all time points.

The fenestration diameter in samples treated with H_2_O_2_ concentrations above 100 µM showed a dose-dependent decrease. In particular, for 500 µM H_2_O_2_ treatment, the fenestration diameter decreased from 167 to 147 nm after 0.5 hours and later increased to 200 nm after 1 hour (Figure 3G). A similar trend was observed for fenestration diameter distribution width which initially decreased after 0.5 hours and then increased after 1 hour for concentrations of 200–500 µM H_2_O_2_. In similar ranges of concentrations of 100–1000 µM, after 2 hours of treatment, it was impossible to distinguish fenestrations in SEM due to disturbed cell membranes (Figure 3D).

**FIGURE 4:**
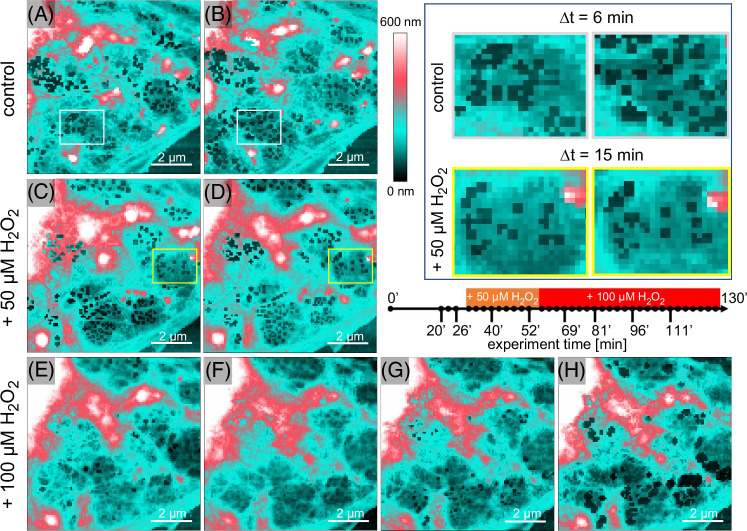
Dynamic defenestration and refenestration in live LSEC treated with hydrogen peroxide. Presented images (A–H) correspond with consecutive time points (20'-111')﻿ indicated on a timeline.﻿ The selected LSEC periphery with several sieve plates was scanned with AFM for 30 minutes, revealing normal fenestration dynamics (white squares, A/B). After treatment with 50 µM H_2_O_2_, the process of reduction of fenestration dynamics (yellow squares, C/D) and reduction of fenestration number was observed. The addition of H_2_O_2_ to a total concentration of 100 µM resulted in further gradual closing of all fenestrations in the following ~20 minutes and reopening in the following 30 minutes. Image size, resolution, and scanning speed were adjusted to obtain a final acquisition of 4 minutes per frame, each represented as a single point on the timeline. Colors correspond to the height of the sample (0–600 nm), with dark teal indicating the substrate, light teal showing a flat cell body, and white/pink indicating a cell body with height above ~500 nm where fenestrations usually cannot be formed. Image size and resolution: 8.5 × 8.5 μm, 100 × 100 pixels) (scale bar = 2 μm). The whole ﻿130 minutes long experiment is﻿ presented as Supplementary video SV1. Abbreviation: AFM, atomic force microscopy.

The combination of initial defenestration followed by reopening of fenestrations and both time-dependent and dose-dependent changes in fenestration diameters suggest dynamic response of LSEC morphology to hydrogen peroxide–induced ROS. To better understand this effect in temporal resolution, we used atomic force microscopy for live imaging of LSEC treated with H_2_O_2_ (Figure 4).

Highly dynamic fenestrations and sieve plates changing their size and position over time were detected initially. After treatment with 50 µM H_2_O_2_, we observed a reduction in fenestration number, confirming the SEM data from Figure 3. Moreover, the reduction of fenestration dynamics occurred nearly immediately after the injection of the agent into cell culture. The challenge with an additional 50 µM H_2_O_2_ resulted in gradual fenestration closing within 20 minutes. Still, fenestration-associated cytoskeleton ring structure could be easily distinguished (Figures 4E, F) while fenestrations remained closed and cell membranes fused. During the following 30 minutes, we observed a gradual reopening of fenestrations. Reopened fenestrations quickly increased their dimensions often exceeding 300 nm. Newly formed fenestrations did not migrate within the cell and remained arrested at the same position. After an additional 30 minutes, we observed cell flattening at the peripheries with numerous fenestrations and gaps (Supplementary video SV1). Similar results were reproduced in 2 independent experiments (Supplemental Figure S3, http://links.lww.com/HC9/B868), confirming the SEM results.

**Supplementary Video V1:** Dynamic defenestration and refenestration in live LSEC treated with hydrogen peroxide. The selected area between two LSEC was scanned with AFM for 30 min revealing normal fenestration dynamics. After treatment with 50 μM H2O2 the reduction of fenestration dynamics and fenestration number were observed. Later, the addition of H2O2 to a total concentration of 100 μM resulted in further gradual closing of all fenestrations within ~20 min and reopening in the following 30 min. Imaging parameters: image size (8.5×8.5 μm), resolution (100×100 pixels), scanning speed (4 minutes per frame), total time of the experiment (130min). Selected frames from the experiment are presented in Figure 4.”

### ROS-induced changes in LSEC cytoskeleton

**FIGURE 5:**
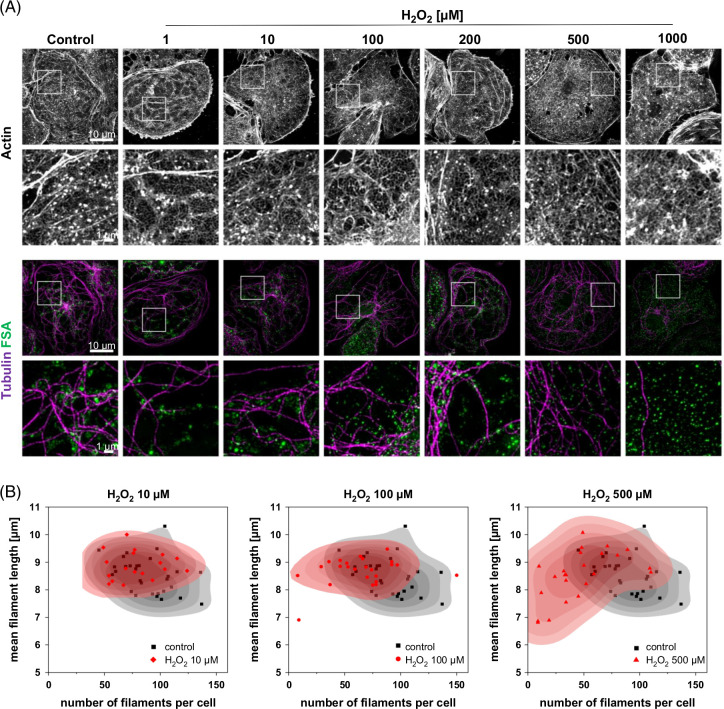
Changes in LSEC cytoskeleton after treatment with hydrogen peroxide. (A) Representative SIM projection images of actin and tubulin cytoskeleton in rat LSEC treated with hydrogen peroxide. Cells were treated for 2 hours with H_2_O_2_ with FSA-AF488 added for the last 15 minutes and, after fixation, stained with phalloidin-AF555 and anti-α-tubulin-AF647 antibody. The presented cells were selected based on the positive FSA signal indicating that the cell could still perform endocytosis. For H_2_O_2_ treatment above 50 μM, only a fraction of the LSEC population can still take up FSA, as shown in overview images in Figure 1D. The bottom rows show high-magnification images (10 × 10 µm) of the areas indicated, and the top rows show low-magnification images (40.96 µm × 40.96 µm). (B) Quantitative analysis of tubulin filaments in rat LSEC treated with 0–500 μM H_2_O_2_​​​​​​. The mean filament length and number of filaments per cell were analyzed from randomly selected widefield fluorescent images. Each point represents a single cell; untreated control is marked with black, while treatment groups are shown in red. Abbreviations: FSA, formaldehyde-treated serum albumin; SIM, structured illumination microscopy.

In LSEC, both fenestrated morphology and scavenging functions are closely connected with the cytoskeleton. Therefore, we studied the changes in actin and tubulin under the influence of H_2_O_2_ using super-resolution optical nanoscopy to visualize the fine structure of the cytoskeleton (Figure 5A). In high concentrations (>100 µM), hydrogen peroxide disrupted the tubulin structure with numerous cells presenting a visibly reduced number of tubulin fibers (Supplemental Figure S4A, http://links.lww.com/HC9/B868). The quantitative analysis of the tubulin in H_2_O_2_-treated LSEC (Figure 5B) confirmed both the decrease in the number of tubulin fibers as well as the shift toward shorter mean filament length. Moreover, in the affected cells, microtubules seem to lose their characteristic organization—fibers emerging from the centrosome and surrounding sieve plates, and independent unconnected fibers were observed. For high concentrations of H_2_O_2_, FSA was observed evenly scattered within the cell body and no longer followed tubulin fibers suggesting disturbed transportation of the endocytic vesicles. No significant changes or stress fiber formation were observed in the actin cytoskeleton (Figure 5). Undisturbed actin mesh and regular fenestration-associated cytoskeleton were observed in both treated and untreated cells.

### ROS-depletion system in LSEC

**FIGURE 6:**
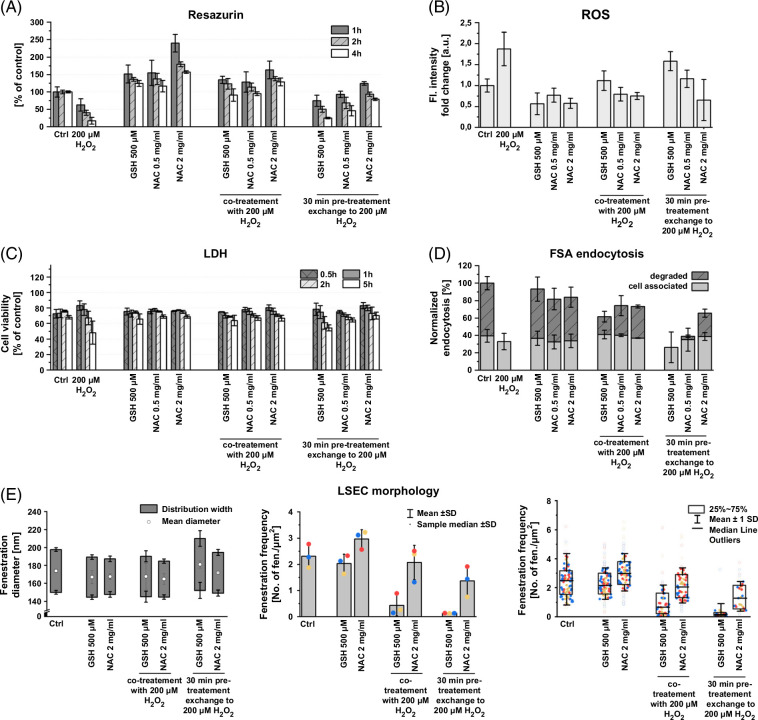
The effect of ROS-inducing versus ROS-depleting agents on rat LSEC viability, functions, and morphology. Cells were treated with ROS-inducing agents, hydrogen peroxide, or ROS-depleting agents: GSH or NAC. For cotreatments, samples were simultaneously treated with both hydrogen peroxide and GSH/NAC at the starting point of the assays. For pretreatments, samples were treated with GSH/NAC for 30 minutes, then rinsed with fresh media and treated with H_2_O_2_ at the starting point of the assays. The functional (A) and structural (C) viability were studied using resazurin and LDH release assays, respectively; (B) intracellular ROS levels were assessed using a fluorescence-based ROS detection assay after 1 hour of treatment and (D) endocytic activity was measured with radiolabeled FSA-based scavenging assay during 2 hours of treatment. (E) LSEC morphology was quantitatively assessed using SEM images. Colors represent data from separate bioreplicates. Abbreviations: FSA, formaldehyde-treated serum albumin; GSH, glutathione; LDH, lactase dehydrogenase; NAC, *n*-acetyl cysteine; ROS, reactive oxygen species; SEM, scanning electron microscopy.

To better understand the LSEC defense mechanisms against ROS, the effects of ROS-depleting agents were studied. A cotreatment/pretreatment with GSH (500 µM) and NAC (0.5–2 mg/mL) together with 200 µM H_2_O_2_ was used, and viability, internal ROS levels, and scavenging activity were assessed (Figure 6). The H_2_O_2_ concentration was selected based on results from the previous sections and showed a clear time-dependent reduction in functional and structural viability, a nearly 2-fold increase in intracellular ROS, and reduced endocytic activity with completely inhibited FSA degradation.

ROS-depleting agents were tested independently and showed no effect on structural viability (in LDH release assay) or endocytic activity (Figures 6C, D). GSH and NAC decreased the intracellular ROS and significantly increased the reduction potential of the cells as shown in the functional viability resazurin assay (statistical results in Supplemental Table S5 and S7, http://links.lww.com/HC9/B868). Mitochondrial activity as a reduction potential of the cell was increased when exposed to ROS-depleting agents which provide cells with additional reducing power. LSEC morphology was not affected by GSH, while NAC treatment led to a 30% increase in fenestration numbers without changes in fenestration diameters (Figure 6E).

In comparison with the hydrogen peroxide challenge alone, simultaneous treatment with GSH or NAC showed a similar reduction of negative ROS effects. Cotreatment with NAC (both 0.5 and 2 mg/mL) almost completely mitigates the decrease in viability and intracellular ROS production and keeps the endocytic activity above 80% of control. GSH cotreatment, although reducing the effect of H_2_O_2_, still led to an increase in intracellular ROS and a decrease in endocytic activity, especially degradation. The cell-associated fractions exhibited no significant differences in comparison to the control. For the degraded fractions, treatment with 200 µM H_2_O_2_ together with all pretreatments and the GSH cotreatment was significantly different from the control, indicating no reduction in the effect of H_2_O_2_ (Figure 6D; Supplemental Table S8, http://links.lww.com/HC9/B868).

To exclude the effects of any direct interaction between GSH/NAC and hydrogen peroxide, sequential treatment was used. In samples with anti-ROS pretreatment, only the higher concentration of NAC prevented negative ROS effects, while the lower concentration of NAC showed a reduction in intracellular ROS and LDH release but still led to a decrease in endocytic activity. Pretreatment with GSH had no ROS-reducing effect, and after following treatment with hydrogen peroxide, an increase in intracellular ROS and a decrease in viability and endocytic activity were observed (Figures 6B–D).

Based on the previous results, only the higher, 2 mg/mL, concentration of NAC was used to study LSEC morphology (Figure 6E). Fenestration diameter distributions were not affected by cotreatments with GSH/NAC and H_2_O_2_. Two hours of treatment with 200 µM H_2_O_2_ led to complete disruption of cell membranes with no cells remaining for morphological fenestration analyses (Figure 3). In samples pretreated with GSH, but not NAC, an increase in mean fenestration diameter and in fenestration diameter distribution width was observed. Fenestration frequency in samples with NAC cotreatment (2.1 ± 0.5) remained on the same level as in control (2.3 ± 0.3), while in pretreatment samples a slight decrease (1.4 ± 0.5) occurred. In cells cotreated with GSH, significant defenestration was observed; however, in comparison with treatment with hydrogen peroxide alone, the cell membranes remained intact in the majority of cells. Pretreatment with GSH led to significant, nearly complete loss of fenestrations, fenestration enlargement, and gap formation (statistical analysis in Supplemental Table S9, http://links.lww.com/HC9/B868).

## DISCUSSION

Cellular oxidative stress is defined as an imbalance between the production of ROS and the reduction by various antioxidants. Elevated ROS correlates with metabolic syndrome, where ROS levels trend upward with elevated BMI, and are reduced with weight loss.^22^ A high fat/western diet causes an increase in ROS levels in both rat and mouse models.^23^,^24^ In the liver, excessive ROS formation can occur in states of inflammation,^25^ by activated KCs and HSCs, or in hepatocytes during intoxication events. LSECs were not reported to contribute to liver ROS production, but due to their placement, LSECs can be exposed to high oxidative stress from both exogenous oxidants in the portal vein and other hepatic cells. Hydrogen peroxide is widely used to induce ROS formation in studying oxidative stress.^26^,^27^ Physiologically, hydrogen peroxide is a product of mitochondrial metabolism and various H_2_O_2_ secretion levels were reported in the livers of different species.^28^

Although LSECs represent only about 15%–20% of the total number of liver cells and 3% of the liver volume, they remain understudied, particularly in the context of oxidative stress compared to hepatocytes, which constitute about 60% of the liver cell population and 80% of liver volume. This disparity highlights a need to focus on LSEC, as their expansive surface area plays a pivotal role in modulating liver physiology and pathology under oxidative conditions. In this study, we used H_2_O_2_ to generate intracellular ROS formation in LSEC in vitro and observed a clear pattern in the effects on cell viability, scavenging function, and morphology for a wide range of hydrogen peroxide concentrations. Intracellular ROS levels slightly increased in the concentration of 5–100 µM but in a non–dose-dependent manner. Only for high concentrations of H_2_O_2_ above 100 µM, a dose-dependent increase in ROS was detected.

### Scavenging

LSECs are the main component of the body’s scavenger system, removing several grams of waste macromolecules per day from circulation.^9^,^29^ Our results show that ROS can irreversibly reduce the endocytic activity even after very short exposure times. The cell’s degrading ability is affected first, while with increasing concentrations also, the cell-associated fraction decreases. Redox homeostasis has been shown to regulate lysosomal function^30^ and increased ROS can hamper lysosomal function directly by preventing acidification and destabilizing lysosomal structure or indirectly by interfering with transport of endosomes. The tubulin network creates a highway for the transportation of endocytosed ligands to the lysosomes in LSEC, and we observed deteriorated tubulin cytoskeleton, however, only for high concentrations of H_2_O_2_. For concentrations of 50–200 μM, the fraction of LSECs unable to perform endocytosis efficiently is increasing, rather than a decrease in endocytic activity per cell. These results suggest that for low concentrations of ROS, the lysosomal function is primarily affected, while with the increasing dose amount of ROS, disruption of tubulin contributes to the reduction of scavenging function.

The impaired clearance by the scavenging system was found in aging,^31^ as well as other liver diseases^32^ and could be in part due to sustained inflammation and ROS generation by immune cells such as KCs. This impairment of waste clearance is linked with liver disease–related kidney injury,^33^ and could present an approach to prevention or amelioration through antioxidants. The interconnected nature of scavenging cells causes failures in one type, especially LSEC, cascade over to other systems (such as splenic and liver clearance of dead cells, cell remnants, bacteria, and senescent erythrocytes) which depend on the same receptors;^34^,^35^ thus, impairments to the liver would impact spleen and bone marrow uptake as well by reducing the capacity of the overall system.^36^ Our data indicates NAC as an opportune candidate for cases with abnormally high oxidative stress.

### Morphology

We found that in vitro exposure to hydrogen peroxide reduces fenestration number in LSEC in a dose-dependent manner during the first 0.5 hours of exposure, but fenestrations reopen after about 1 hour before the membrane disintegrates toward 2 hours of treatment. These findings correlate with the findings of Cogger et al.,^37^ where rat livers were perfused with 70 and 700 µM H_2_O_2_. In their study, a decrease in the number of fenestration and thickening of the endothelium was observed after 10 minutes, similar to our results from live imaging with atomic force microscopy (Figure 4). Moreover, the defenestration centers we observed destabilize the LSEC structure and make them more prone to damage, which may explain the gap formation in the liver perfusion model. In the report of Martinez et al.,^38^ the generation of endogenous H_2_O_2_ was related to a faster rate of defenestration of rat LSEC in culture. LSECs were cultured in 20% versus 5% O_2_, and increased levels of H_2_O_2_ were measured after 24/48 hours in high-oxygen conditions which correlated with lower porosity. LSEC defenestration and gap formation were also observed in vivo in mice with elevated oxidative stress associated with a high-fat western diet.^24^

Furthermore, in live LSEC imaging under the influence of H_2_O_2_, we observed the closing of fenestrations without disrupting the underlying fenestration-associated cytoskeleton and loss of the dynamics. We previously showed similar effects in LSECs challenged with antimycin A^39^—a mitochondrial cytochrome c reductase inhibitor known for increasing ROS production and with diamide^40^—a known cytoskeletal drug that disrupts spectrin. The complete structure of LSEC fenestrations is not yet fully understood, but these results suggest that the ROS-induced defenestration is related to the oxidation and destabilization of spectrin, and possibly other proteins that connect the cell membrane to fenestra-associated cytoskeleton. Protein disulfide isomerase A1 was recently identified to regulate fenestration dynamics and its inhibition led to significant cytoskeleton-independent reduction of fenestration number.^41^ Although LSEC fenestrated morphology is related to the actin cytoskeleton,^10^ we observed no effect of H_2_O_2_ on the cytoskeleton, indicating that the H_2_O_2_ effect is independent of actin.

### Viability

After exposure to H_2_O_2_, LSECs first lose their reducing equivalents before cell death as shown by resazurin assay and LDH release, respectively. The effect is both dose-dependent and time-dependent, with cell death showing clear threshold effects. The reducing equivalents are depleted first, comparing the same time points and concentrations, before the cells proceed toward cell death, suggesting that LSEC survival depends on reducing equivalents to counteract the ROS. Similar observations have been made for ROS-mediated conditions such as sinusoidal obstruction syndrome^42^ or DILI.^43^ Intriguingly, the viability and reducing potential of LSECs, when compared with morphology, indicate that the cells close their fenestrations as reducing equivalents are being consumed. This can potentially be a hepatoprotective mechanism against the sudden increase in ROS-generating factors in the environment, especially considering the first-pass effect that exposes LSECs to higher than systemic plasma level concentrations of potentially harmful substances absorbed through the gastrointestinal tract. The reopening of fenestration could delay the exposure of hepatocytes until the dilution of the stressors within systemic circulation. Nevertheless, more research is needed to explain this phenomenon as well as confirm it in vivo.

### NAC/GSH

A GSH-based defense system in LSEC has been previously described to play a protective role in hepatic ischemia-reperfusion injury (HIRI), virus infections, and drug-induced liver toxicity.^11^ We observed that the effects of hydrogen peroxide–induced ROS can be mitigated by simultaneous treatment with GSH or GSH precursor—NAC. The results with pretreatments, where only NAC but not GSH reduced the negative H_2_O_2_ effects, suggest that LSECs do not store excess amounts of GSH but rather can readily produce it in the occurrence of oxidative stress conditions provided with the fuel such as, for example, NAC. Similar data linking depletion of endogenous GSH with exacerbated cytotoxicity and the addition of exogenous GSH with reduced toxicity were noted by Deleve et al.^44^ Similarly, in chemically induced sinusoidal obstruction syndrome models, ROS-related damage of LSECs can be prevented by cotreatment with GSH.^45^ This suggests that the cells can survive for as long as they have reducing equivalents to counteract the ROS. The expenditure of reducing equivalents thus eventually leads to cell damage and death, if more than what the cell can produce/regenerate. The intracellular GSH levels in LSECs are much lower in comparison with hepatocytes, 0.5–1.5 fmol/cell and 17–50 fmol/cell, respectively,^11^,^44^ making LSECs more sensitive to oxidative stress. The limited reducing capability of LSEC can explain our observation of a non–dose-dependent increase in intracellular ROS levels for lower H_2_O_2_ concentration of 5–100 µM, which suggests that the cell can successfully neutralize ROS until some threshold level. Only for high concentrations of H_2_O_2_ (>100 µM), a dose-dependent increase in ROS was detected.

NAC is typically used as a hepatoprotective agent to prevent, for example, HIRI^46^ or DILI,^47^ especially acetaminophen overdose.^48^ In both HIRI and DILI prevention, the reduction of oxidative stress and ROS production was shown to play a crucial role. Moreover, ROS are required for KC proinflammatory/antigen-presenting activity,^49^ and NAC, as well as other antioxidants, decrease LPS-induced KC activation and TNF-alpha secretion.^50^ Our results with NAC pretreatment being protective against ROS suggest that the reduced LSEC toxicity is crucial to reducing overall hepatic toxicity in both HIRI/DILI. As shown by Cogger and colleagues, acute exogenous oxidative stress leads to gap formation in LSEC and disruption of the endothelial layer, leading to further exposure of hepatocytes to the portal blood. Reduction of ROS-related toxicity in LSEC can help avoid further exposure of hepatocytes and alterations of the space of Disse preventing the toxicity for the whole organ.

The translational potential of this study may be reduced by using animal-derived cells instead of human LSECs, representing its primary limitation. We are aware of the availability of commercial human LSECs; however, the number of cells required for the designed experiments makes the costs unreasonably high. Moreover, we intend to use only primary not passaged LSECs as these cells are known for losing their characteristic features, namely fenestrations and endocytic activity, in culture.^38^ We hypothesize that the ROS scavenging system in LSECs, as described here, would be conserved among the species and intend to verify it in our future studies.

## CONCLUSIONS

Hydrogen peroxide–induced intracellular ROS formation was used to study oxidative stress effects on rat LSECs in vitro. ROS irreversibly reduced endocytic/scavenging function, potentially due to disrupted tubulin cytoskeleton at high levels. ROS caused a dose-dependent reduction in LSEC fenestrations within 0.5 hours, followed by reopening before membrane disintegration around 2 hours. ROS-induced LSEC defenestration is possibly related to oxidation and destabilized fenestration ultrastructure, independent of actin. LSECs lose reducing equivalents before undergoing cell death upon H_2_O_2_ exposure, suggesting fenestration closure as a potential hepatoprotective mechanism against sudden ROS increases also suggesting antioxidants as a potential therapeutic approach. NAC and GSH mitigate H_2_O_2_-induced ROS effects in LSECs, with NAC pretreatment being more effective, indicating LSECs can readily produce GSH under oxidative stress if provided with precursors such as NAC. NAC’s protective effects against ROS-mediated LSEC toxicity could contribute to its hepatoprotective role in conditions like ischemia-reperfusion injury and DILI, by preventing further hepatocyte exposure and alterations in the space of Disse.

## Data Availability

The data sets used and/or analyzed during the current study and not provided in the manuscript/supplemental information are available from the corresponding author upon reasonable request. Conceptualization: Karolina Szafranska, Christopher Holte, and Larissa D. Kruse. Data curation: Christopher Holte, Larissa D. Kruse, Karolina Szafranska, Wolfgang Hübner, Bartlomiej Zapotoczny, and Eike C. Struck. Formal analysis: Eike C. Struck, Larissa D. Kruse, and Christopher Holte. Funding acquisition: Thomas Huser and Bartlomiej Zapotoczny. Investigation: Christopher Holte, Larissa D. Kruse, Karolina Szafranska, Jasmin Schürstedt, Wolfgang Hübner, and Bartlomiej Zapotoczny. Methodology: Christopher Holte, Larissa D. Kruse, Karolina Szafranska, Jasmin Schürstedt, Wolfgang Hübner, Bartlomiej Zapotoczny, and Eike C. Struck. Supervision: Karolina Szafranska. Visualization: Christopher Holte, Larissa D. Kruse, Karolina Szafranska, Wolfgang Hübner, and Bartlomiej Zapotoczny. Writing—original draft: Christopher Holte, Larissa D. Kruse, and Karolina Szafranska. Writing—review and editing: all authors. The authors thank Randi Olsen and Tom-Ivar Eilertsen from the Advanced Microscopy Core Facility at UiT for electron microscopy expertise and Professor Peter McCourt for the linguistic revision of the manuscript. This study was supported by the European Union’s European Innovation Council (EIC) PATHFINDER Open Programme, project DeLIVERy, under grant agreement No. 101046928 and Hop-On Facility HORIZON-WIDERA program associated with DeLIVERy. The authors have no conflicts to report.
